# Supplementary material for: Moiré-Induced Transport in CVD-Based Small-Angle Twisted Bilayer Graphene
Source: Nano Lett. 2022 Jul 1;22(13):5252–9. doi: 10.1021/acs.nanolett.2c01114 (PMC9284678; doi:10.1021/acs.nanolett.2c01114)
Supplement: Supplementary file 1 — nl2c01114_si_001.pdf [file nl2c01114_si_001.pdf]

Supporting Information for

Moiré-induced transport in CVD-based small-angle twisted bilayer graphene

Giulia Piccinini[1,2], Vaidotas Mišeikis[2,3], Pietro Novelli[4], Kenji Watanabe[5], Takashi Taniguchi[6], Marco Polini[7,3], Camilla Coletti[2,3,*], Sergio Pezzini[8,**]

[1]*NEST, Scuola Normale Superiore, Piazza San Silvestro 12, 56127 Pisa, Italy*
[2]*Center for Nanotechnology Innovation @NEST, Istituto Italiano di Tecnologia, Piazza San Silvestro 12, 56127 Pisa, Italy*
[3]*Graphene Labs, Istituto Italiano di Tecnologia, Via Morego 30, 16163 Genova, Italy*
[4]*Istituto Italiano di Tecnologia, Via Melen 83, 16152 Genova, Italy*
[5]*Research Center for Functional Materials, National Institute for Materials Science, 1-1 Namiki, Tsukuba, 305-0044, Japan*
[6]*International Center for Materials Nanoarchitectonics, National Institute for Materials Science, 1-1 Namiki, Tsukuba, 305-0044, Japan*
[7]*Dipartimento di Fisica, Università di Pisa, Largo Bruno Pontecorvo 3, 56127 Pisa, Italy*
[8]*NEST, Istituto Nanoscienze-CNR and Scuola Normale Superiore, Piazza San Silvestro 12, 56127 Pisa, Italy*

* camilla.coletti@iit.it

** sergio.pezzini@nano.cnr.it

Experimental Methods

CVD synthesis of SLG arrays:

The CVD synthesis of graphene single-crystals arrays is performed in an Aixtron BM Pro cold-wall. Cr-patterned copper foils are used as growth substrate, as described in Ref. [S1].

Raman spectroscopy:

Raman spectroscopy is carried out with a Renishaw InVia spectrometer equipped with a 532 nm laser, using a 100x objective and 0.2 mW laser power (giving a ~500 nm spot size).

Device fabrication:

The hBN/TBG/hBN stacks are processed using standard e-beam lithography, combined with reactive ion etching and metal evaporation. We first fabricate edge contacts by e-beam patterning of PMMA, followed by etching of the heterostructure in CF_4/O_2 and self-aligned metallization (Cr/Au 5/50 nm). Then we fabricate a top-gate (Cr/Au 5/50 nm), ensuring to avoid short circuit with contacts and exposed graphene edges. Finally, we etch the stack

to define a 6-contacts Hall bar, using the physical mask provided by the metallic top gate, in combination with additional e-beam-patterned PMMA arms.

Low-temperature magnetotransport:

The devices are measured in a ^{4}He cryostat with a 12 T superconducting coil. We use standard AC lock-in detection (13 Hz) with a constant current (20 nA) through the device. The top and bottom gates are biased via two independent channels of a source-meter and controlled via a home-built acquisition program.

Assembly of a second SA-TBG sample

As discussed in the main text, the target angle chosen for our study on SA-TBG is θ = 2.5°. The controllability on the twist angle is affected by several factors, namely:

i) the uncertainty in the rotation of the transfer stage. Our setup, shown in Figure S1a ensures an instrumental error of ~0.01° when employing the high-precision actuator.

ii) the tendency of TBG to relax toward smaller twist angle during van der Waals assembly. Due to this phenomenon, it is a common practice in the TBG community to assemble the graphene flakes at a slightly (i.e., few tens of degrees) larger angle with respect to the experimental target [S2]. The sample reported in the main manuscript relaxed from 2.5° to 2.4°. A second assembly targeted at 2.5° – that we describe in detail below – results in a Raman response indistinguishable from the device in the main text (see Figure S1e). However, we find a more severe relaxation when targeting a smaller twist angle (see next Section).

iii) strain induced by interface contaminants. We do avoid areas with a high concentration of bubbles. Our fabrication is targeted exclusively to flat parts of the samples, which are identified by combining optical microscopy and atomic force microscopy. Assembly at higher temperatures and/or post assembly annealing might help increasing the yield of flat areas, however at risk of increasing the tendency toward stronger relaxation of the twist angle.

The reproducibility of the procedure described in the main text is tested with the assembly of a second SA-TBG sample targeted at 2.5°. The hBN flake used as pick-up carrier and top encapsulant is shown in Figure S1b. The two CVD-grown graphene crystals on SiO_2/Si selected for the assembly are shown in Figure S1c: the two hexagonally-shaped graphene clearly share the same crystallographic orientation. In Figure S1d we show the same crystals after performing – in this order – pick-up of SLG 1, rotation of the stage, pick-up of SLG 2. The shape of the hBN flake (black continuous line) remains negatively imprinted on the crystals, meaning the that graphene has been picked-up. Additional damage to the crystals is caused by unintentional pick-ups from hBN flakes nearby the selected one (some of which are visible in Figure 1b). By using a straight edge of the hBN as a

reference, the rotation performed within the two successive pick-ups can be clearly appreciated (see white dotted lines). After completing the assembly, we identify flat areas that we characterize with Raman spectroscopy. In Figure S1e we show a representative spectrum from the second sample (SA-TBG_SI), along with the spectrum from the sample discussed in the main text (SA-TBG_main). The characteristic 2D peak of SA-TBG is perfectly reproduced, with the two Lorentzian components (light blue and light red peaks) positioned within ±1 cm^{-1} in the two spectra.

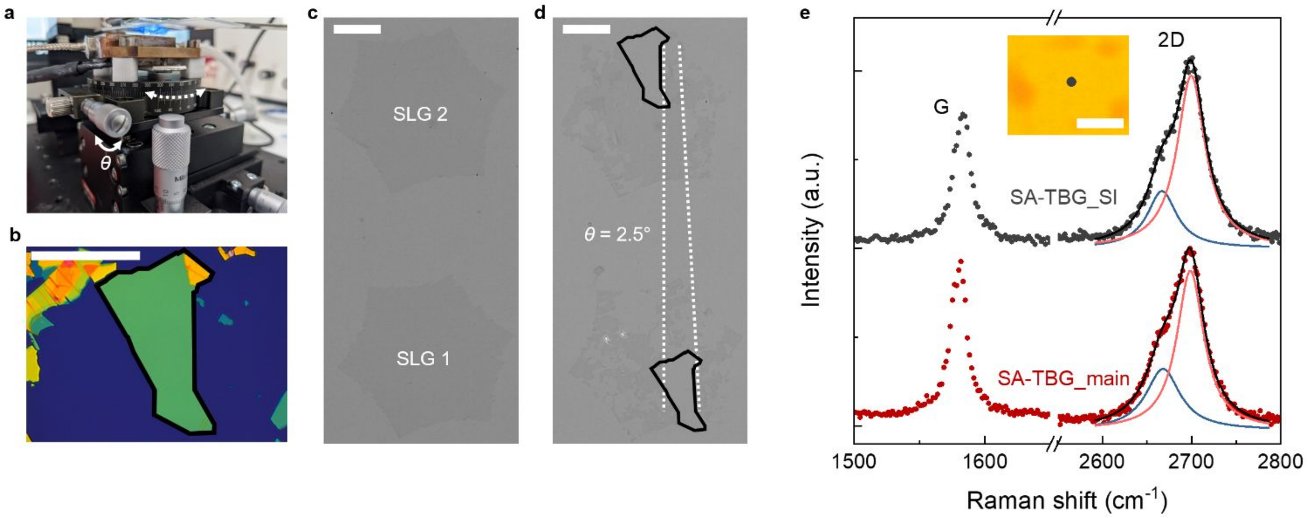

Figure S1. (a) Close-up image of the setup used for van der Waals assembly. The rotation of the stage (white dotted arrow) is controlled via a high-precision actuator (white continuous arrow). (b) Optical microscopy image of the hBN flake used as top layer for the assembly of a second SA-TBG sample. The contour of the flake is marked by the black continuous line. (c) CVD-grown aligned SLG crystals selected for the assembly. The black-and-white colors are chosen to enhance the visibility of graphene on top of SiO_2/Si. (d) The same SLG crystals after the pick-up process. The two contact positions of the hBN flake from panel b are marked by the black contour. The white dotted lines are aligned to a straight edge of the hBN flake at the two contact positions, to highlight the controlled 2.5° rotation between the successive pickups. (e) Raman spectrum of the second SA-TBG sample (dark grey), compared to the sample presented in the main text (dark red). A double-Lorentzian fit to the 2D peak is shown for both spectra. Inset: optical microscopy image of the second hBN-encapsulated SA-TBG. The dark grey spot indicates the point where the dark grey spectrum in the main panel is acquired. Scale bars are 50 µm in panels b, c, d; 2.5 µm in panel e inset.

CVD-based bilayer graphene with sub-MA twisting

Following the same procedure described in the main text for 2.4°-TBG, we assembled a third sample targeting the sub-MA regime ($\lesssim 1°$). Dual-gated transport measurements reveal a smaller twist angle (θ~0.53°), close to the so-called second MA [S2], due to uncontrolled relaxation at the assembly stage, similar to Ref.[S2].

Figure S2 shows a representative Raman spectrum of the sub-MA sample (black curve), with 2.4°-TBG and Bernal-stacked bilayer graphene (BLG) taken as references. Consistent with Ref.[S3], the Raman spectrum in the sub-MA TBG appears similar to that of BLG. However, two main differences can be detected: (i) the full width at half maximum of the G peak is ~12 cm^{-1} in BLG, while it is ~17 cm^{-1} in sub-MA TBG; (ii) the 2D peak of BLG clearly shows four sharp Lorentzian components, while a broad asymmetric peak is observed for sub-MA TBG. Both these features are reported in Ref.[S3], and are uniformly observed over the area chosen for device fabrication.

In Figure S3 we present low temperature transport measurements on the sub-MA TBG sample, performed in two-terminal configuration. The inset of Figure S3 shows a color map of the resistance as function of the top and back-gate potentials. Besides the CNP, two additional high-resistance features are visible. By inspecting the resistance along a line-cut perpendicular to the CNP, three equally spaced peaks are evident (Figure S3 main panel). We identify the additional satellite peaks as corresponding to full filling of the moiré bands at Γ_s. The total charge density at the peaks n_s (calculated from the sum of the gate voltages weighted with the respective gate capacitance) allows to estimate the moiré periodicity according to $n_s = \frac{8}{\sqrt{3}\lambda^2}$, and thereby the twist angle. For this device we obtain θ = (0.53±0.02)°, indicating proximity to the second MA [S2].

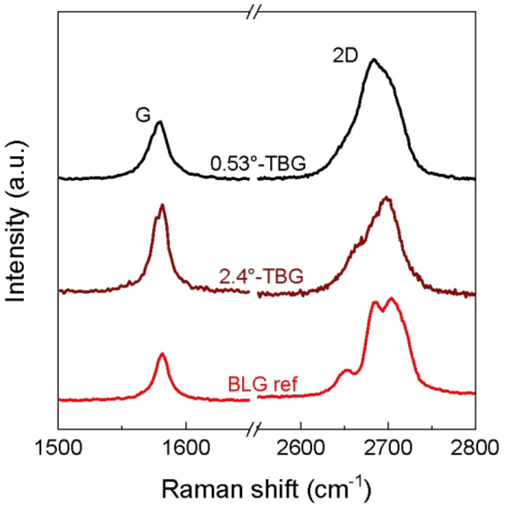

Figure S2. Representative Raman spectra of 0.53°-TBG (black), 2.4°-TBG (dark red), and BLG (red).

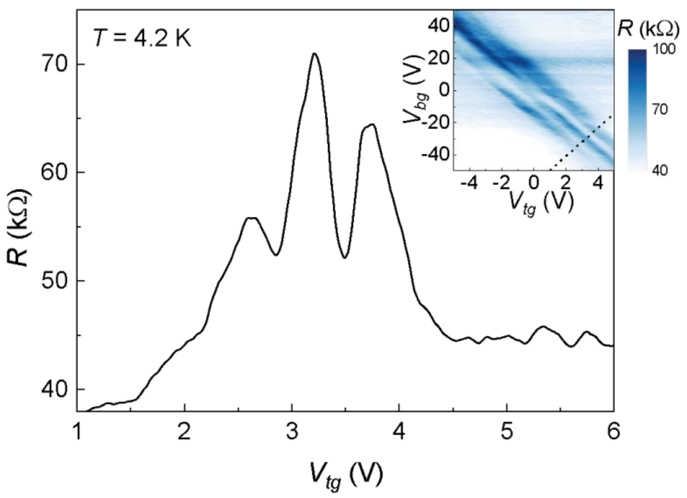

Figure S3. Resistance of sub-MA TBG as a function of the top-gate voltage, measured in a two-terminal configuration along the dashed line in the inset. Inset: color map of the two- terminal resistance as a function of the gate voltages.

Dependence of the CNPs splitting on Fermi velocity and interlayer capacitance

In the manuscript, we estimated the Fermi velocity (v_F) and the interlayer capacitance (C_{gg}) of SA-TBG from the LLs trajectories as a function of the gate voltages (Figure 2a). Using the obtained v_F and C_{gg} values, we then compute the positions of the layers' CNPs and compare them to a map of the zero-field conductivity (Figure 2e). In Figure S4 we show how the CNPs splitting depends on the v_F and C_{gg}. The trajectories are highly sensitive to these parameters: the splitting increases by reducing the Fermi velocity (Fig.S4a), as well as by reducing the interlayer capacitance (Fig.S4b).

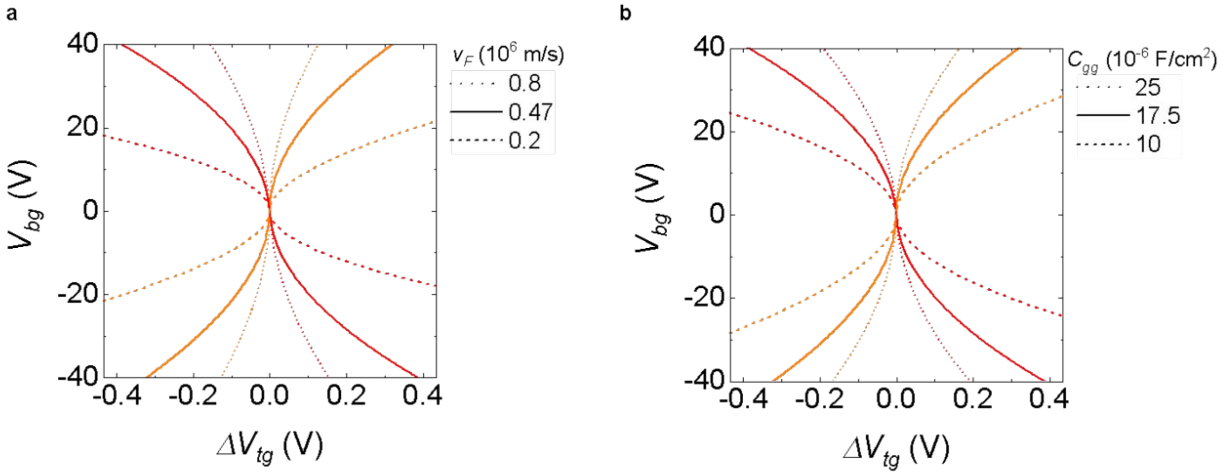

Figure S4. Calculated charge neutrality points of the upper (orange) and lower (red) layer as a function of top-gate voltage relative to the sample CNP, and back-gate voltage. The CNPs evolution varying the Fermi velocity is shown in (a), varying the interlayer capacitance in (b). Solid lines are CNPs calculated with the optimized parameters $v_F = 0.47 \times 10^6$ m/s and $C_{gg} = 17.5 \times 10^{-6}$ F/cm^2.

Additional References

[S1] V. Miseikis *et al.*, Deterministic patterned growth of high-mobility large-crystal graphene: A path towards wafer scale integration, *2D Mater.* **4**, 021004 (2017).

[S2] X. Lu *et al.*, Multiple flat bands and topological Hofstadter butterfly in twisted bilayer graphene close to the second magic angle, *Proc. Natl. Acad. Sci. USA* **118**, e2100006118 (2021).

[S3] C. Gadetha *et al.*, Localization of lattice dynamics in low-angle twisted bilayer graphene, *Nature* **590**, 405–409 (2021).
